# A novel deep learning radiopathomics model for predicting carcinogenesis promotor cyclooxygenase-2 expression in common bile duct in children with pancreaticobiliary maljunction: a multicenter study

**DOI:** 10.1186/s13244-025-01951-5

**Published:** 2025-03-27

**Authors:** Hui-min Mao, Jian-jun Zhang, Bin Zhu, Wan-liang Guo

**Affiliations:** 1 Department of Radiology, Children’s Hospital of Soochow University, Suzhou, China; 2 Department of Neonatal Surgery, Xuzhou Children’s Hospital, Xuzhou, China; 3 Department of Interventional Therapy, Xuzhou Children’s Hospital, Xuzhou, China

**Keywords:** Radiomics, Deep learning, Pathomics, Cyclooxygenase-2, Pancreaticobiliary maljunction

## Abstract

**Objectives:**

To develop and validate a deep learning radiopathomics model (DLRPM) integrating radiological and pathological imaging data to predict biliary cyclooxygenase-2 (COX-2) expression in children with pancreaticobiliary maljunction (PBM), and to compare its performance with single-modality radiomics, deep learning radiomics (DLR), and pathomics models.

**Methods:**

This retrospective study included 219 PBM patients, divided into a training set (*n* = 104; median age, 2.8 years, 75.0% females) and internal test set (*n* = 71; median age, 2.2 years, 83.1% females) from center I, and an external test set (*n* = 44; median age, 3.4 years, 65.9% females) from center II. Biliary COX-2 expression was detected using immunohistochemistry. Radiomics, DLR, and pathomics features were extracted from portal venous-phase CT images and H&E-stained histopathological slides, respectively, to build individual single-modality models. These were then integrated to develop the DLRPM, combining three predictive signatures. Model performance was evaluated using AUC, net reclassification index (NRI, for assessing improvement in correct classification) and integrated discrimination improvement (IDI).

**Results:**

The DLRPM demonstrated the highest performance, with AUCs of 0.851 (95% CI, 0.759–0.942) in internal test set and 0.841 (95% CI, 0.721–0.960) in external test set. In comparison, AUCs for the radiomics, DLR, and pathomics models were 0.532–0.602, 0.658–0.660, and 0.787–0.805, respectively. The DLRPM significantly outperformed three single-modality models, as demonstrated by the NRI and IDI tests (all *p* < 0.05).

**Conclusion:**

The multimodal DLRPM could accurately and robustly predict COX-2 expression, facilitating risk stratification and personalized postoperative management in PBM. However, prospective multicenter studies with larger cohorts are needed to further validate its generalizability.

**Critical relevance statement:**

Our proposed deep learning radiopathomics model, integrating CT and histopathological images, provides a novel and cost-effective approach to accurately predict biliary cyclooxygenase-2 expression, potentially advancing individualized risk stratification and improving long-term outcomes for pediatric patients with pancreaticobiliary maljunction.

**Key Points:**

- Predicting biliary COX-2 expression in pancreaticobiliary maljunction (PBM) is critical but challenging.
- A deep learning radiopathomics model achieved high predictive accuracy for COX-2.
- The model supports patient stratification and personalized postoperative management in PBM.

**Graphical Abstract:**

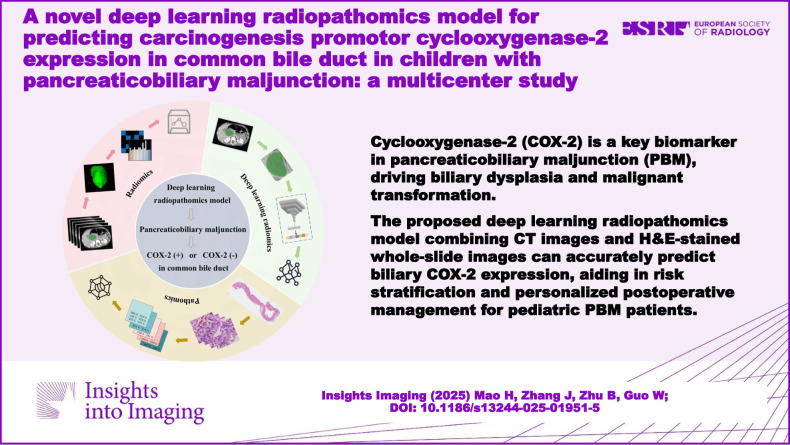

## Introduction

Pancreaticobiliary maljunction (PBM) is a congenital defect that is characterized by an abnormal junction of pancreatic and bile ducts outside of the duodenal wall, usually forming a long common channel [1]. PBM is more prevalent in Asia, with an estimated incidence 100 to 1000 times higher than in other regions of the world [2]. This anatomical anomaly disrupts the regulation of the pancreaticobiliary junction by the sphincter of Oddi, typically resulting in reflux of pancreatic juice into the common bile duct (CBD) [3]. PBM is considered to involve a hyperplasia–dysplasia–carcinoma sequence caused by chronic inflammation induced by constant reflux, which significantly increases the risk of biliary cancers [1, 3–5]. Even after surgical treatment, patients with PBM remain at risk of developing biliary malignancies [6, 7], necessitating close follow-up and personalized risk-stratified management.

Cyclooxygenase-2 (COX-2), a key protein in prostaglandin metabolism, has been implicated in inflammation and carcinogenesis [8, 9]. In patients with PBM, overexpression of COX-2 is observed from the early stage of biliary injury, which stimulates angiogenesis, promotes dysplasia of biliary mucosal epithelium, and increases the risk of transformation from dysplasia to carcinoma [10, 11]. Previous studies have demonstrated that COX-2 inhibitors, such as nonsteroidal anti-inflammatory drugs, can effectively prevent carcinogenesis in PBM [12, 13]. Accordingly, accurate prediction of COX-2 expression could contribute to the postoperative tailored interventions, including the selective use of COX-2 inhibitors to halt the progression of precancerous biliary tract lesions or adjusting surveillance intervals based on individual risk profiles.

Clinically, immunohistochemistry (IHC) is the current conventional approach for COX-2 assessment. However, its application to every patient with PBM is challenging because of its high economic and time expenses, as well as the requirement for additional tissue specimens. Therefore, it is necessary to explore a universally applicable method to conveniently and accurately evaluate COX-2 expression in PBM.

Recently, machine learning and deep learning (DL) techniques have made remarkable progress in medicine, especially the combination of artificial intelligence and medical images, showing outstanding capabilities in disease diagnosis, molecular typing, and survival and prognosis assessment [14–16]. Radiomics, extracting quantitative features from radiological images, offers macroscale insights into tissue characteristics such as texture, shape, and spatial heterogeneity [17]. This approach has proven feasibility in assessing PBM-related diseases [18, 19]. Similarly, pathomics involves the extraction of high-dimensional microscopic features from whole slide images (WSIs)—high-resolution digital scans of histopathological slides. By leveraging DL algorithms, pathomics based on hematoxylin and eosin (H&E)-stained WSIs has shown promise in predicting molecular markers, such as human epidermal growth factor receptor 2, TP53, and KRAS genotypes [20–22]. When radiomics and pathomics are combined, the resulting multiscale radiopathomics provides complementary and cohesive information, thereby significantly enhancing the predictive power and robustness of existing models [23–25]. Despite its promising potential, there have been no studies using radiopathomics to predict COX-2 expression in PBM.

Thus, we hypothesized that a multimodal model integrating radiological and H&E-stained histopathological images could accurately predict COX-2 expression in children with PBM. This study aimed to develop and validate a deep learning radiopathomics model (DLRPM) for the prediction of COX-2 expression in pediatric patients with PBM using CE-CT images and H&E-stained WSIs.

## Methods

This study was approved by the Institutional Review Board of each participating hospital (no. 2021CS146), and the requirement for informed consent was waived because of its retrospective nature.

### Study patients

**Fig. 1:**
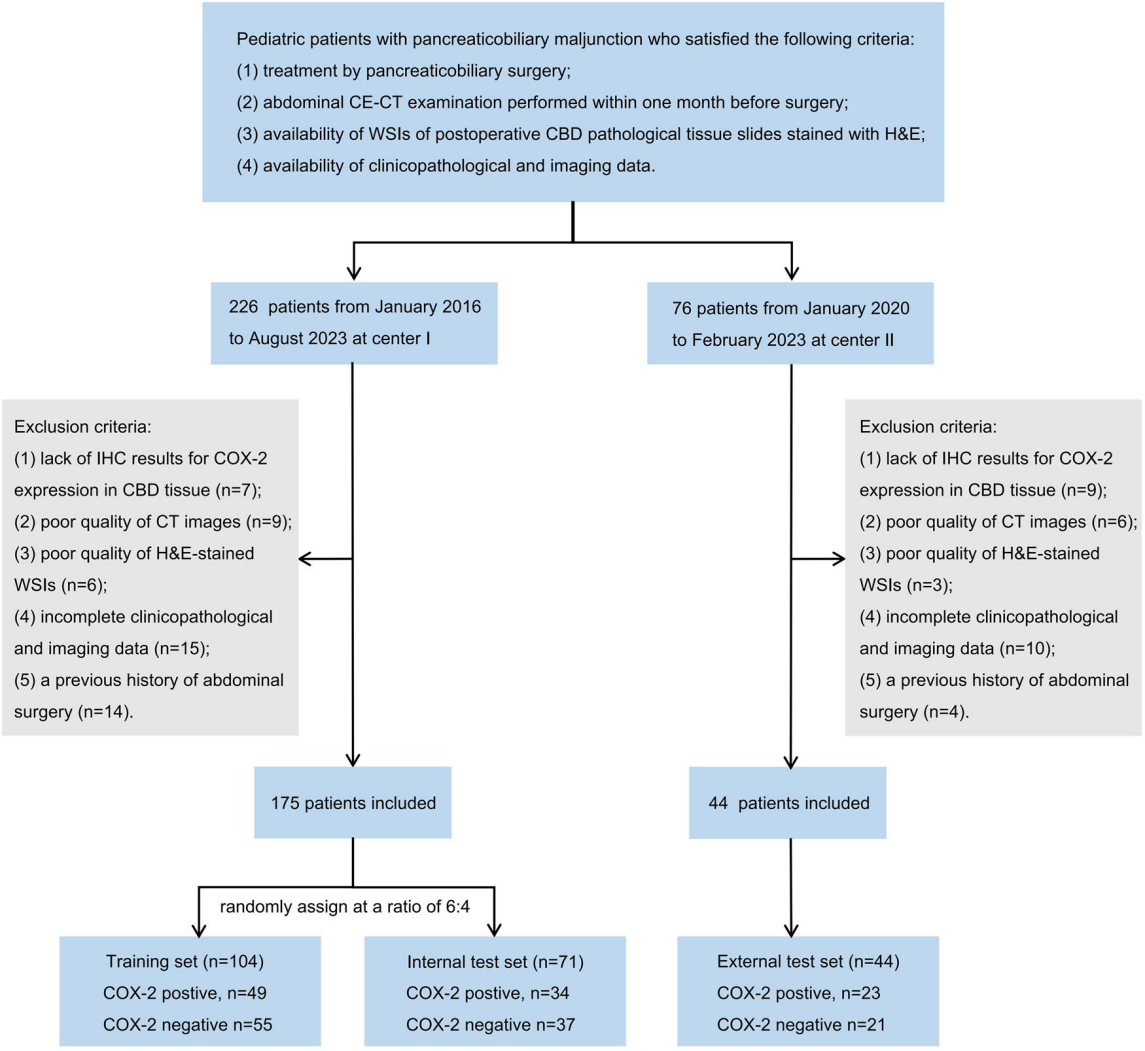
Flowchart of patient selection from two medical centers. CBD, common bile duct; CE-CT, contrast-enhanced computed tomography; COX-2, cyclooxygenase-2; H&E, hematoxylin and eosin; IHC, immunohistochemistry; WSI, whole slide imaging

A total of 219 pediatric patients with PBM from two centers between January 2016 and August 2023 were retrospectively collected. The patients from center I (*n* = 175) were randomly divided into a training set (*n* = 104; median age, 2.8 years, 75.0% females) and an internal test set (*n* = 71; median age, 2.2 years, 83.1% females) at a 6:4 ratio. The patients from center II (*n* = 44; median age, 3.4 years, 65.9% females) were included as an external test set. The inclusion/exclusion criteria are detailed in Supplementary Method S1, and the patient selection flowchart is shown in Fig. 1. COX-2 expression in CBD tissue was assessed using IHC (Supplementary Method S2).

**Fig. 2:**
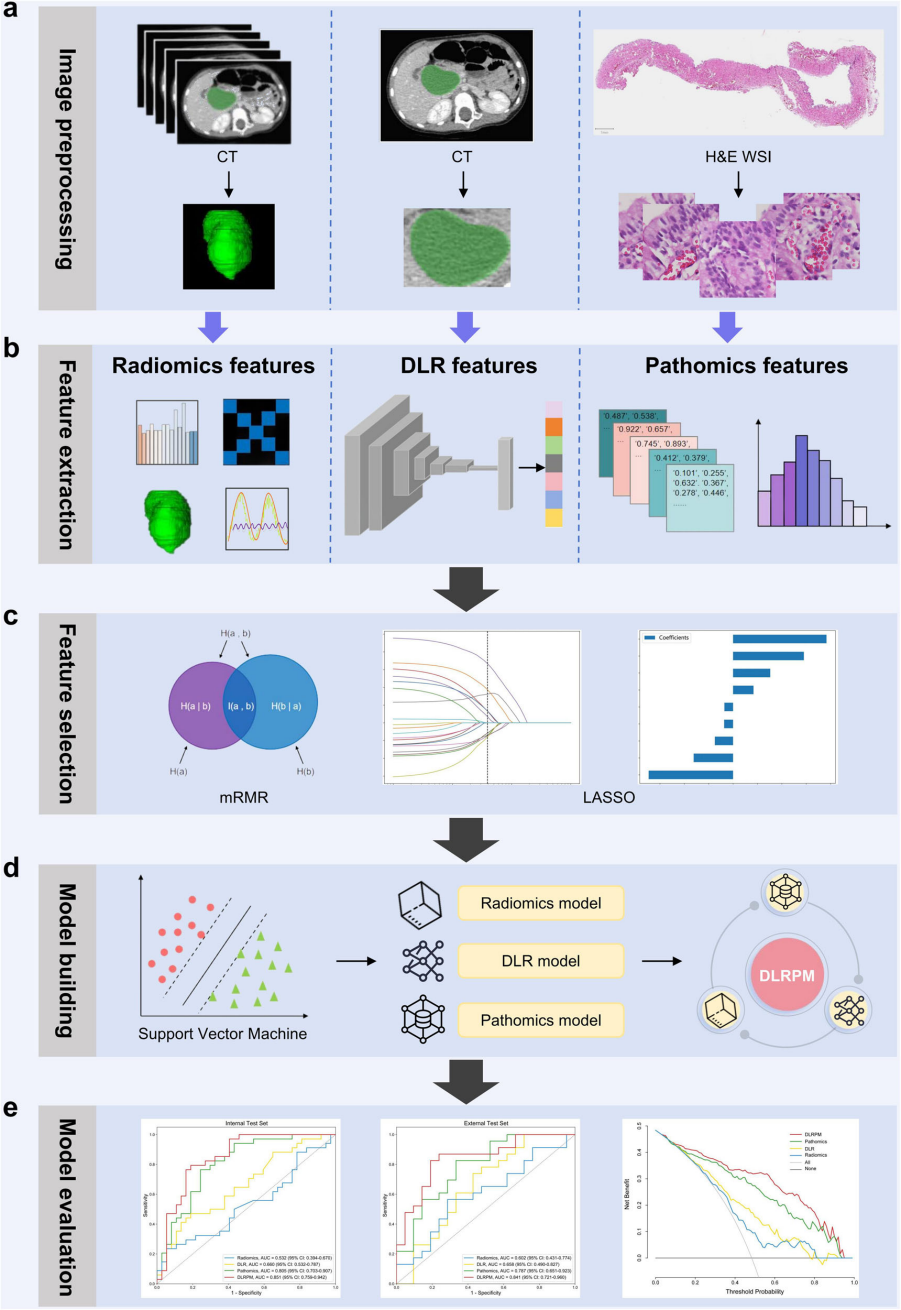
Workflow of the study. **a** Images of portal venous-phase computed tomography (CT) and hematoxylin and eosin (H&E)-stained whole slide imaging (WSI) were acquired and segmented. **b** Radiomics, and deep learning radiomics (DLR), and pathomics features were extracted separately. **c** Feature selection was based on the minimum redundancy maximum relevance (mRMR) and least absolute shrinkage and selection operator (LASSO). **d** Radiomics, DLR, and pathomics models were constructed, and a deep learning radiopathomics model (DLRPM) was further developed by integrating these three single-modality signatures. **e** The performance of all established models for predicting cyclooxygenase-2 status in pediatric patients with pancreaticobiliary maljunction was evaluated

Baseline clinical characteristics, including demographics, clinical symptoms, laboratory testing, and imaging reporting, were recorded (Table S1). The overall workflow of the study is illustrated in Fig. 2.

### CT examination and image preprocessing

All patients underwent abdominal contrast-enhanced computed tomography (CE-CT) examination within one month before surgery. Portal venous-phase CT images were retrieved from Picture Archiving and Communication Systems for further analysis. Detailed imaging parameters, including CT systems, tube voltage, rotation time, and contrast agent protocols, are outlined in Table S2. Image preprocessing is presented in Supplementary Method S3.

### Radiomics and deep learning radiomics (DLR) feature extraction

On axial portal venous-phase CT images, three-dimensional regions of interest (ROIs) of the entire visible CBD were manually delineated across all of the axial contiguous slices (Supplementary Method S3). Radiomics features were extracted from the segmented three-dimensional ROIs using Pyradiomics Module (Supplementary Method S4), and DLR features were extracted from the two-dimensional rectangular ROIs using ResNet50 model (Supplementary Method S5).

### WSI acquisition and preprocessing

All H&E-stained CBD slides were scanned at 40× objective lens to obtain WSIs, which were then cropped into small patches (details provided in Supplementary Method S6).

Two patch-level prediction strategies were implemented: one using all patches, representing a general approach often used in weakly supervised pathomics research [26–28], and the other focusing on mucosal patches identified with the ResNeXt101 model (Supplementary Method S7), representing a strategy that targets relevant areas for COX-2 expression. Six convolutional neural networks (CNNs) were applied to both patch-level strategies (Supplementary Method S8). The pathomics workflow of this study is illustrated in Fig. S1.

### Pathomics feature extraction

For pathomics features, we initially compared six CNNs under two patch-level prediction strategies, using the area under the curve (AUC) as the primary performance metric. The CNN with the highest AUC was selected for subsequent pathomics feature extraction (Supplementary Method S8). To derive WSI-level pathomics features, the patch likelihoods predicted by the selected CNN were aggregated using a multi-instance learning framework (Supplementary Method S9). This approach effectively integrated the dispersed patch-level predictions into a holistic representation of the pathological image for each sample.

### Radiopathomics feature selection

Prior to feature selection, three types of features were normalized using the Z-score method to ensure comparability across features with diverse scales. After that, two methods, namely, the minimum redundancy maximum relevance (mRMR) and least absolute shrinkage and selection operator (LASSO), were used to select the most representative features in the training set. Further, t-distributed stochastic neighbor embedding (t-SNE) algorithm (perplexity = 30, iterations = 1000) was employed to visualize the distribution of the selected features in two-dimensional space and assess their clustering patterns.

### Model development

Following feature screening, three types of optimal features were employed to develop three distinct single-modality prediction models. The predictive probabilities derived from each model output were obtained as pathomics signature, radiomics signature, and DLR signature. Subsequently, the DLRPM was developed by integrating three single-modality signatures. In addition, the following dual-modality prediction models were constructed: the Rad-DLR model, integrating radiomics and DLR signatures; the Rad-Path model, integrating radiomics and pathomics signatures; and the DLR-Path model, integrating DLR and pathomics signatures.

All models (single-modality, DLRPM, and dual-modality models) were developed using the support vector machine. Hyperparameter tuning was conducted in the training set using five-fold cross-validation and the Grid-Search algorithm. Regularization was controlled through the “C” parameter, which ranged from 0.01 to 10, to balance model complexity and mitigate overfitting risks. All developed models were validated in internal and external test sets to assess their generalizability and robustness.

### Model evaluation

The performances of all established models were measured using the receiver operator characteristic (ROC) analysis and AUC. The Delong test was used to compare AUC values. Optimal cutoff values, derived from the maximum Youden index in the training set, were utilized for calculating accuracy, sensitivity, specificity, positive predictive value (PPV), and negative predictive value (NPV). The Youden index, defined as the sum of sensitivity and specificity minus one, was employed to determine the optimal threshold for balancing diagnostic performance. By maximizing the Youden index, the threshold optimizes the trade-off between minimizing false negatives (missed diagnoses) and false positives (misdiagnoses). To compare the performance of the DLRPM with that of the single-modality models, net reclassification index (NRI) and integrated discrimination improvement (IDI) were implemented to assess the improvement in predictive accuracy. NRI quantifies the improvement in correct classification, while IDI evaluates the change in average predicted probabilities. The Hosmer-Lemeshow test and calibration curve were used to assess the goodness of fit of the DLRPM. Decision curve analysis (DCA) was conducted to compare the net benefit difference between the DLRPM and the single-modality models across various threshold probabilities (ranging from 0 to 1), aiming to ascertain their respective clinical value in different clinical decision-making scenarios.

### Statistical analysis

All statistical analyses were conducted using R software (version 4.2.1), Python (version 3.9.12), and IBM SPSS software (version 26.0). Categorical variables were analyzed using the chi-square (χ^2^) test or Fisher’s exact test. For continuous variables, comparisons between two groups were conducted using either independent sample *t*-test or Mann–Whitney *U* test, whereas one-way analysis of variance or Kruskal–Wallis test was employed for comparisons among more than two groups. A two-tailed *p*-value < 0.05 indicated a statistically significant difference.

Feature selection was performed using mRMR and LASSO analyses, utilizing the “mRMR” and “glmnet” R packages, respectively. t-SNE visualization was conducted with the Python “sklearn.manifold.TSNE”. Model performance metrics and Delong test were conducted using “sklearn.metrics” and “pyroc” Python libraries, respectively. NRI and IDI were calculated with custom R scripts. DCA was performed using “rmda” R package.

## Results

### Patient characteristics

The baseline clinical characteristics are presented in Table 1 and Supplementary Table S1. The training set comprised 104 patients (median age, 2.8 years; 78 (75.0%) females), the internal test set included 71 patients (median age, 2.2 years; 59 (83.1%) females), and the external test set contained 44 patients (median age, 3.4 years; 29 (65.9%) females). The rate of COX-2 positive status was comparable across the three sets, namely, 47.1% (49/104) in the training set, 47.9% (34/71) in the internal test set, and 52.3% (23/44) in the external test set. There were no statistically significant differences in baseline information between COX-2 positive and COX-2 negative patients.

**Table 1:** Baseline characteristics of patients categorized by COX-2 status in the training, internal test, and external test sets

Characteristics	Training set (*n* = 104)		*p*-value	Internal test set (*n* = 71)		*p*-value	External test set (*n* = 44)		*p*-value
	COX-2 positive (*n* = 49)	COX-2 negative (*n* = 55)		COX-2 positive (*n* = 34)	COX-2 negative (*n* = 37)		COX-2 positive (*n* = 23)	COX-2 negative (*n* = 21)	
Age (years), median (IQR)	2.1 (1.4–3.8)	3.0 (1.7–5.6)	0.061	2.1 (1.0–4.9)	2.3 (1.1–4.1)	0.952	3.8 (1.4–5.4)	2.8 (1.2–4.7)	0.391
Female, No. (%)	39 (79.6)	39 (70.9)	0.307	29 (85.3)	30 (81.1)	0.636	16 (69.6)	13 (61.9)	0.592
Abdominal pain, No. (%)	29 (59.2)	37 (67.3)	0.392	22 (64.7)	25 (67.6)	0.799	14 (60.9)	13 (61.9)	0.944
Jaundice, No. (%)	14 (28.6)	11 (20.0)	0.307	9 (26.5)	9 (24.3)	0.835	6 (26.1)	6 (28.6)	0.853
Fever, No. (%)	9 (18.4)	12 (21.8)	0.662	4 (11.8)	7 (18.9)	0.405	4 (17.4)	3 (14.3)	< 0.999
Vomiting, No. (%)	28 (57.1)	28 (50.9)	0.524	17 (50.0)	18 (48.6)	0.909	15 (65.2)	14 (66.7)	0.919
Abdominal mass, No. (%)	3 (6.1)	3 (5.5)	< 0.999	4 (11.8)	3 (8.1)	0.906	2 (8.7)	1 (4.8)	< 0.999
IVa of Todani classification, No. (%)	26 (53.1)	22 (40.0)	0.182	19 (55.9)	19 (51.4)	0.702	10 (43.5)	7 (33.3)	0.490
Diameter of CBD (mm), median (IQR)	16.5 (16.5–32.7)	22.5 (15.1–30.2)	0.740	26.5 (18.5–47.3)	23.8 (15.5–34.2)	0.362	22.4 (19.8–43.3)	22.6 (18.0–30.3)	0.286
Biliary stones, No. (%)	27 (55.1)	23 (41.8)	0.176	20 (58.8)	17 (45.9)	0.278	12 (52.2)	14 (66.7)	0.329
Peribiliary fluid collection, No. (%)	19 (38.8)	13 (23.6)	0.095	11 (32.4)	13 (35.1)	0.804	4 (17.4)	7 (33.3)	0.223
Elevated WBC count, No. (%)	14 (28.6)	17 (30.9)	0.795	12 (35.3)	13 (35.1)	0.989	7 (30.4)	3 (14.3)	0.359
Elevated AST, No. (%)	15 (30.6)	17 (30.9)	0.974	10 (29.4)	10 (27.0)	0.823	7 (30.4)	11 (52.4)	0.139
Elevated ALT, No. (%)	18 (36.7)	21 (38.2)	0.879	16 (47.1)	17 (45.9)	0.925	13 (56.5)	10 (47.6)	0.555
Elevated GGT, No. (%)	29 (59.2)	27 (49.1)	0.303	22 (64.7)	26 (70.3)	0.617	16 (69.6)	16 (76.2)	0.622
Elevated TBil, No. (%)	18 (36.7)	19 (34.5)	0.816	8 (23.5)	13 (35.1)	0.284	6 (26.1)	6 (28.6)	0.853
Elevated serum amylase, No. (%)	13 (26.5)	12 (21.8)	0.575	5 (14.7)	6 (16.2)	0.861	6 (26.1)	2 (9.5)	0.302

*ALT* alanine aminotransferase, *AST* aspartate aminotransferase, *CBD* common bile duct, *COX-2* cyclooxygenase-2, *GGT* gamma-glutamyl transferase, *IQR* interquartile range, *TBil* total bilirubin, *WBC* white blood cell

### Radiopathomics feature extraction and selection

Based on portal venous-phase CT images, a total of 1834 radiomics features and 2048 DLR features were extracted for each participant. Additionally, pathomics feature extraction in this study involved three key steps. First, the pathological structure classification model exhibited outstanding performance in identifying patches with the mucosal region (AUCs > 0.950), enabling the model to classify unannotated patches (Supplementary Result S1 and Fig. S2). After that, the performance of two patch-level prediction strategies was compared. As shown in Supplementary Fig. S3, the second strategy, which focused on patches with the mucosal region using DenseNet121 model, achieved the highest patch-level prediction performance. This approach attained AUC values of 0.710 (95% confidence interval (CI), 0.706–0.714) and 0.644 (95% CI, 0.636–0.652) in the internal and external test sets, respectively (Supplementary Result S2). Finally, leveraging this DenseNet121 model, a total of 202 WSI-level pathomics features were extracted for each participant through multi-instance learning.

**Fig. 3:**
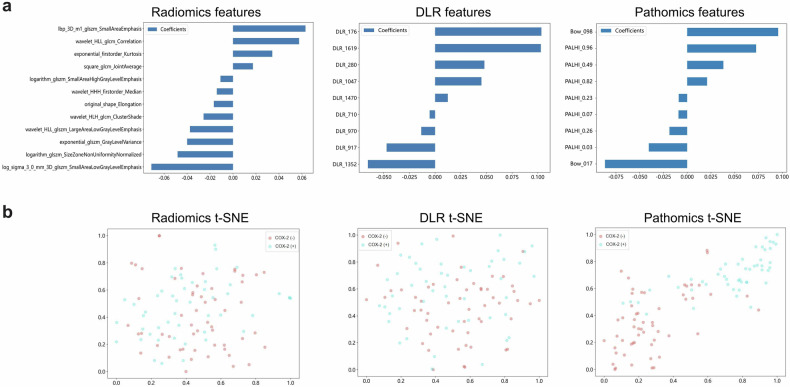
Feature selection and visualization. Three types of feature selection by the least absolute shrinkage and selection operator (**a**) and feature visualization by the t-distributed stochastic neighbor embedding (t-SNE) algorithm (**b**) in the training set. BoW, bag of words; COX-2 (−), cyclooxygenase-2 negative; COX-2 (+), cyclooxygenase-2 positive; DLR, deep learning radiomics; PALHI, patch likelihood histogram

Regarding feature selection, the mRMR algorithm was employed to remove redundant and irrelevant features, retaining the top 20 features from each modality for subsequent LASSO selection. Ultimately, twelve radiomics, nine DLR, and nine pathomics features were selected per patient (Fig. 3a). The t-SNE visualizations of the three feature types illustrated that the pathomics features exhibited superior discriminative capability compared with the radiomics features and DLR features, as evidenced by their distinct separation (Fig. 3b). This suggests that the pathomics features can capture more disease-specific information, thereby offering greater potential for accurate COX-2 prediction.

### Model development and evaluation

**Fig. 4:**
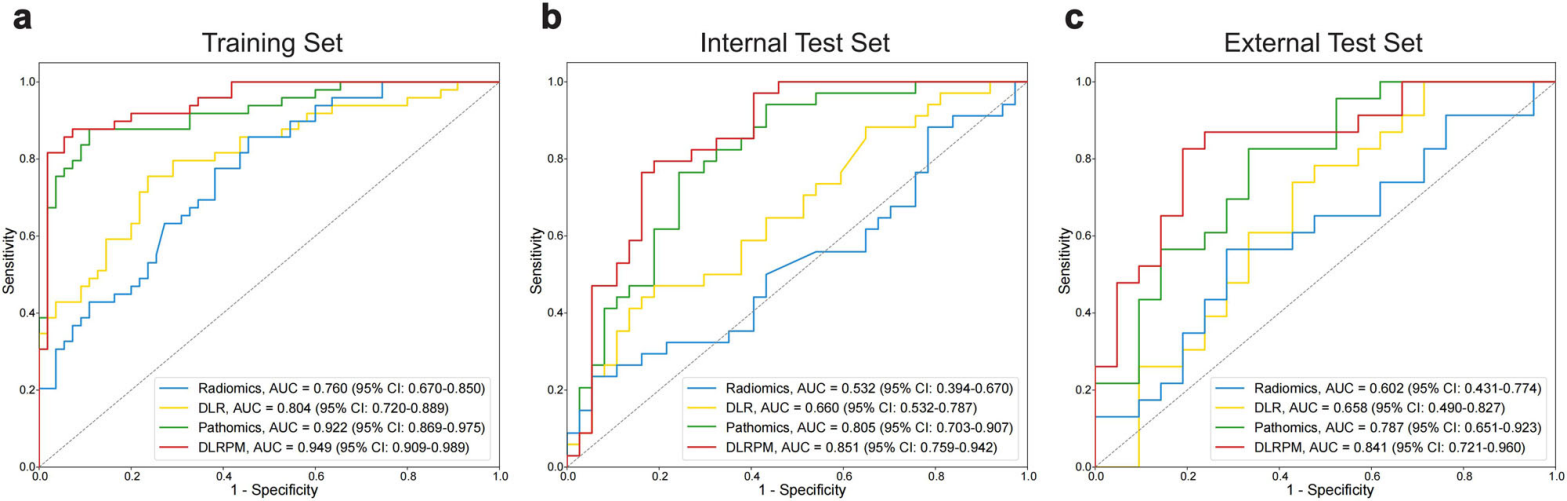
Receiver operating characteristic (ROC) curves of single-modality models and deep learning radiopathomics model (DLRPM). ROC curves for predicting cyclooxygenase-2 status in pediatric patients with pancreaticobiliary maljunction among the radiomics model, deep learning radiomics (DLR) model, pathomics model, and DLRPM in the training set (**a**), internal test set (**b**), and external test set (**c**), respectively. AUC, area under the curve; CI, confidence interval

By integrating the radiomics, DLR, and pathomics signatures, the DLRPM exhibited a good performance in predicting the COX-2 status, with AUC values of 0.851 (95% CI, 0.759–0.942) and 0.841 (95% CI, 0.721–0.960) in the internal and external test sets, respectively (Fig. 4). The DLRPM significantly outperformed the radiomics model (AUC = 0.532 [95% CI, 0.394–0.670; *p* < 0.001] in the internal test set; 0.602 [95% CI, 0.431–0.774; *p* = 0.028] in the external test set) and the DLR model (AUC = 0.660 [95% CI, 0.532–0.787; *p* = 0.005] in the internal test set; and 0.658 [95% CI, 0.490–0.827; *p* = 0.041] in the external test set) (Table 2 and Fig. S4). The pathomics model achieved higher AUC values (0.805 [95% CI, 0.703–0.907] and 0.787 [95% CI, 0.651–0.923] in the internal and external test sets, respectively) compared with the other single-modality models (Table 2). In the test sets, despite not meeting the significance threshold in the DeLong test, the performance of the pathomics model was still lower than that of the DLRPM. The improvements in predicting COX-2 expression by the DLRPM over the single-modality models were further confirmed by NRI tests (all NRI > 0, *p* < 0.05; Table S3) and IDI tests (all IDI > 0, *p* < 0.05; Table S4).

**Table 2:** Performances of the predictive models in the training, internal test, and external test sets

Model	AUC (95% CI)	Accuracy	Sensitivity	Specificity	PPV	NPV
Training set						
Radiomics model	0.760 (0.670–0.850)	0.692 (72/104)	0.857 (42/49)	0.545 (30/55)	0.627 (42/67)	0.811 (30/37)
DLR model	0.804 (0.720–0.889)	0.760 (79/104)	0.755 (37/49)	0.764 (42/55)	0.740 (37/50)	0.778 (42/54)
Pathomics model	0.922 (0.869–0.975)	0.885 (92/104)	0.878 (43/49)	0.891 (49/55)	0.878 (43/49)	0.891 (49/55)
DLRPM	0.949 (0.909–0.989)	0.904 (94/104)	0.878 (43/49)	0.927 (51/55)	0.915 (43/47)	0.895 (51/57)
Internal test set						
Radiomics model	0.532 (0.394–0.670)	0.451 (32/71)	0.676 (23/34)	0.243 (9/37)	0.451 (23/51)	0.450 (9/20)
DLR model	0.660 (0.532–0.787)	0.592 (42/71)	0.588 (20/34)	0.595 (22/37)	0.571 (20/35)	0.611 (22/36)
Pathomics model	0.805 (0.703–0.907)	0.704 (50/71)	0.618 (21/34)	0.784 (29/37)	0.724 (21/29)	0.690 (29/42)
DLRPM	0.851 (0.759–0.942)	0.803 (57/71)	0.765 (26/34)	0.838 (31/37)	0.813 (26/32)	0.795 (31/39)
External test set						
Radiomics model	0.602 (0.431–0.774)	0.568 (25/44)	0.739 (17/23)	0.381 (8/21)	0.567 (17/30)	0.571 (8/14)
DLR model	0.658 (0.490–0.827)	0.636 (28/44)	0.739 (17/23)	0.524 (11/21)	0.630 (17/27)	0.647 (11/17)
Pathomics model	0.787 (0.651–0.923)	0.705 (31/44)	0.826 (19/23)	0.571 (12/21)	0.679 (19/28)	0.750 (12/16)
DLRPM	0.841 (0.721–0.960)	0.818 (36/44)	0.870 (20/23)	0.762 (16/21)	0.800 (20/25)	0.842 (16/19)

*AUC* area under the receiver operating characteristic curve, *CI* confidence interval, *DLR* deep learning radiomics, *DLRPM* deep learning radiopathomics model, *NPV* negative predictive value, *PPV* positive predictive value

In addition, the AUCs for the dual-modality models in the two test sets were as follows: 0.647–0.704 for the Rad-DLR model, 0.807–0.810 for the Rad-Path model, and 0.797–0.822 for the DLR-Path model (Fig. S5). These models exhibited lower performance compared to the DLRPM (AUCs: 0.841–0.851), likely due to limited feature interaction and suboptimal integration of complementary information from the different modalities. The variability in performance across the dual-modality models can be attributed to the insufficient combination of modality-specific signatures, a limitation that was effectively addressed by the fully integrated DLRPM. The incorporation of pathomics, reflecting microscopic histopathological characteristics, complemented the macroscopic information from radiomics and DLR, enhancing the DLRPM’s ability to predict COX-2 status.

The optimal cutoff value for each model was determined using the maximum Youden Index in the training set. At the optimized cutoff value of 0.577, the DLRPM presented superior comprehensive performance, with accuracy ranging from 0.803 to 0.818, sensitivity from 0.765 to 0.870, and specificity from 0.762 to 0.838 in the two test sets (Table 2). The waterfall plot displayed the relative distance of each patient’s classification probability from the normalized cutoff value for the DLRPM model, effectively visualizing the distribution of model predictions and immunohistochemically confirmed COX-2 status (Fig. S6a). The calibration curves and Hosmer-Lemeshow tests showed good agreements between the DLRPM prediction and actual observation in all data sets (all *p* > 0.05; Fig. S6b). Additionally, DCA curves demonstrated that the DLRPM yielded a greater net benefit than the single-modality models across a wide range of thresholds in the entire datasets (Fig. S6c).

## Discussion

In this study, we developed radiomics and DLR models based on CT images, as well as a pathomics model derived from H&E-stained slides, to predict COX-2 expression in pediatric patients with PBM. Further, a multimodal model, namely, DLRPM, was established by incorporating three single-modality imaging signatures. The DLRPM demonstrated superior predictive capabilities compared with the single-modality models, along with favorable sensitivity and specificity in both the internal and external test sets.

Previous studies have highlighted the high risks and challenges of managing biliary carcinogenesis in PBM after surgery, which may stem from the accumulation of genetic abnormalities in the remnant bile duct [29–31]. As emphasized by Tsuchida et al [29], it is impractical for patients without cancerous lesions to undergo extensive procedures like pancreaticoduodenectomy or total liver transplantation to remove all biliary ducts. Consequently, preclinical studies and literature reports have suggested that chemoprevention by suppressing angiogenesis with COX-2 inhibitors could be effective in preventing carcinogenesis in PBM [3, 12, 13]. COX-2 plays a critical role in the pathophysiology of numerous diseases, including inflammatory conditions and malignancies, and is strongly associated with increased tumor aggressiveness, metastasis, and poor clinical outcomes [8, 32, 33]. Therefore, establishing a method that could accurately predict the COX-2 expression is of paramount importance for personalized postoperative management in PBM.

Potential applications of radiomics and DLR for predicting molecular alterations have received considerable attention in recent years [34, 35]. For instance, a radiomics model using PET/CT images predicted COX-2 expression in cervical cancer, achieving moderate performance (AUC = 0.748 in the internal test set), but its clinical applicability was limited by small sample sizes and lack of independent validation [36]. In our study, however, relying solely on predefined macrostructural radiomics features from CT images presented challenges in accurately predicting biliary COX-2 expression in PBM. Furthermore, a ResNet50 model was utilized to extract DLR features, and the resulting DLR model showed a modest improvement over the radiomics model. Previous studies have demonstrated that DL can discern complex patterns and capture intricate details from the hidden layers of neural networks without depending on predetermined features, thereby complementing the existing practices of conventional radiomics [37, 38]. Despite this advantage, the performance of DLR model in our study was still not satisfactory. These results suggest that radiology-derived features should be combined with additional auxiliary features to enhance the predictive capacity for COX-2 expression.

Unlike radiographic images, which capture the spatial macrostructure of lesions and surrounding tissues, histopathological images offer detailed microstructural insights into the cellular morphology within localized lesions. Pathomics, derived from features extracted from H&E-stained images using DL algorithms, has proven valuable for assessing various molecular phenotypes, such as TP53 mutations, programmed death ligand-1, and microsatellite instability [21, 39, 40]. In this study, we focused on using patches with mucosal regions, as these regions are particularly relevant to COX-2 expression. By providing insights at the histopathological level, such as inflammation-related changes and mucosal cellular morphology, pathomics could help better discover the underlying biological behaviors associated with COX-2 expression. This may explain why the microscopic pathomics model outperformed the macroscopic radiomics models in predicting COX-2 expression.

While single data modalities can be vulnerable to noise and incomplete information, integrating them with complementary signals from other modalities can significantly enhance analytical robustness, improve predictive accuracy, and provide a more comprehensive understanding of complex phenomena or patterns [41]. Feng et al [23] developed and validated a radiopathomics model to predict pathological complete response in locally advanced rectal cancer. Their multimodal model achieved AUC values ranging from 0.812 to 0.872, surpassing single-modality prediction models. Huang et al [42] reliably predicted molecular subtypes of breast cancer by integrating radiomics and pathomics, distinguishing luminal from non-luminal tumors. A multimodal model combining radiomic signatures, pathomic signatures, and clinical features, was developed to effectively predict postoperative biochemical recurrence in prostate cancer [43]. Similarly, in our study, the DLRPM demonstrated satisfactory performance in predicting the COX-2 expression in the test sets (AUC, 0.841–0.851), outperforming the three single-modality models. Such performance aligns with the general expectations for similar predictive models, where AUC values typically range from 0.810 to 0.900 [23, 42–45]. Moreover, the DLRPM exhibited improved performance over any dual-modality model, indicating the complementarity of multimodal signatures rather than redundant information. These findings suggest that the comprehensive integration of macrostructural radiomics and microstructural pathomics signatures can enhance the prediction of COX-2 expression in children with PBM.

Clinically, by leveraging conventionally available abdominal CE-CT images and H&E-stained slides, the DLRPM can rapidly and accurately predict high-risk patients with COX-2 positivity. This approach eliminates the need for time-consuming and costly IHC examinations. High-risk patients identified by the DLRPM should be closely monitored and actively followed up to prevent malignant transformation. Furthermore, these predictions are particularly relevant in the context of chemoprevention, where the suppression of angiogenesis with a COX-2 inhibitor has been considered effective in preventing carcinogenesis in PBM [13]. Consequently, the DLRPM could serve as a practical and valuable tool for enhancing tailored postoperative guidance and supporting more precise therapeutic decisions in PBM.

The current study has several limitations. First, as this was a retrospective study, results were inevitably influenced by inherent bias. Future research should involve larger, prospectively collected datasets to further validate our findings. Second, variations in CT systems, scan parameters, and H&E-stained tissue sections across different centers might impact the results. Given that, we implemented certain measures for image standardization, including CT image preprocessing (adjusting window level/width and voxel resampling) and pathological image normalization using the Vahadane method, to minimize variability across the datasets. Additionally, future studies could integrate multiphase CT data and additional molecular markers to assess their potential to further improve predictive performance. Finally, the integration of DLRPM into clinical workflows presents challenges, such as computational requirements and clinician training. Addressing these challenges through the development of user-friendly software interfaces and clinician training programs will be essential.

In conclusion, we developed and validated a DLRPM that integrates radiomics, DLR, and pathomics signatures to predict COX-2 expression in pediatric patients with PBM. The proposed multimodal model exhibited promising predictive performance, potentially serving as a valuable tool for facilitating risk stratification of patients and individualized management in PBM.

## Supplementary information

ELECTRONIC SUPPLEMENTARY MATERIAL

## Data Availability

The datasets analyzed during the current study are available from the corresponding author upon reasonable request.

## Abbreviations

AUC: Area under the curve
CBD: Common bile duct
CE-CT: Contrast-enhanced computed tomography
CI: Confidence interval
CNN: Convolutional neural network
COX-2: Cyclooxygenase-2
DCA: Decision curve analysis
DL: Deep learning
DLR: Deep learning radiomics
DLRPM: Deep learning radiopathomics model
H&E: Hematoxylin and eosin
IDI: Integrated discrimination improvement
IHC: Immunohistochemistry
LASSO: Least absolute shrinkage and selection operator
mRMR: Minimum redundancy maximum relevance
NPV: Negative predictive value
NRI: Net reclassification index
PBM: Pancreaticobiliary maljunction
PPV: Positive predictive value
ROC: Receiver operating characteristic
ROI: Region of interest
t-SNE: t-distributed stochastic neighbor embedding
WSI: Whole slide images
