# Supplementary material for: A novel deep learning radiopathomics model for predicting carcinogenesis promotor cyclooxygenase-2 expression in common bile duct in children with pancreaticobiliary maljunction: a multicenter study
Source: Insights Imaging. 2025 Mar 27;16:74. doi: 10.1186/s13244-025-01951-5 (PMC11950503; doi:10.1186/s13244-025-01951-5)
Supplement: Supplementary file 1 — ELECTRONIC SUPPLEMENTARY MATERIAL [file 13244_2025_1951_MOESM1_ESM.pdf]

A novel deep learning radiopathomics model for predicting carcinogenesis promotor cyclooxygenase-2 expression in common bile duct in children with pancreaticobiliary maljunction: a multicenter study

ELECTRONIC SUPPLEMENTARY MATERIAL

Supplementary Methods

Method S1. Patient recruitment

This retrospective multicenter study included 219 pediatric patients with pancreaticobiliary maljunction (PBM) from two centers between January 2016 and August 2023. PBM was diagnosed based on the presence of an abnormally long common channel or an abnormal union between the pancreatic and bile ducts, as confirmed by surgery or imaging examination.[1]

The inclusion criteria were: (1) treatment by pancreaticobiliary surgery; (2) abdominal contrast-enhanced computed tomography (CE-CT) examination performed within one month before surgery; (3) availability of whole slide images (WSIs) of postoperative common bile duct (CBD) pathological tissue slides stained with hematoxylin and eosin (H&E); and (4) availability of clinicopathological and imaging data. The exclusion criteria were: (1) lack of immunohistochemistry (IHC) results for cyclooxygenase-2 (COX-2) expression in CBD tissue; (2) poor quality of CT images or H&E-stained WSIs; (3) incomplete or missing clinicopathological and imaging data; and (4) a previous history of abdominal surgery.

Method S2. Assessment of COX-2 expression

Paraffin-embedded sections of CBD tissue from all of the included subjects were obtained from the department of pathology in the two centers. IHC staining was conducted on 3-μm-thick sections using rabbit anti–human COX-2 polyclonal primary antibody (Abcam, UK; 1:100 dilution). Intensity of COX-2 immunostaining was assessed across four grades as follows: grade 0, indicating no staining; grade 1, weak staining; grade 2, moderate staining; and grade 3, strong staining. When the percentage of stained cells with grade 2 or 3 exceeded 30%, the

sample was considered COX-2 positive, whereas when it fell below 30%, it was deemed COX-2 negative.[2] COX-2 expression was evaluated by consensus of two experienced pathologists who were blinded to the participants' details.

Method S3. CT image preprocessing and segmentation

Reconstructed thin-slice portal venous-phase CT images were retrieved from Picture Archiving and Communication Systems (PACS) for further evaluation. Image standardization was implemented to minimize variability across the datasets sourced from the different centers and to ensure the generation of suitable inputs for quantitative radiomics feature computation. This standardization procedure involved preprocessing the CT images by setting the window level to 50 Hounsfield units (HU) and the window width to 400 HU, followed by image resampling to achieve a voxel spacing of 1 × 1 × 1 mm^3.

Three-dimensional (3D) regions of interest (ROIs) of the CBD across all of the axial contiguous slices were manually delineated by a radiologist (Radiologist A, with 3 years of experience in abdominal imaging) using the ITK-SNAP software (version 3.8.0; http://www.itksnap.org). After one month, 30 randomly selected cases from the training set underwent repeat 3D ROI segmentation, independently conducted by Radiologist A and another radiologist (Radiologist B, with 10 years of experience in abdominal imaging). Both radiologists were blinded to the study data. The intra- and inter-observer reproducibility of radiomics features was evaluated, and only features with intra- and inter-observer correlation coefficients > 0.85 were retained for subsequent feature selection.

Method S4. Radiomics feature extraction

Radiomics features of the 3D ROIs representing the CBD were extracted using Pyradiomics Module (http://pyradiomics.readthedocs.io),[3] with gray level discretization performed at a fixed bin width of 25 HU. A total of 1834 quantitative features from seven categories were extracted for every patient: (1) 14 shape-based features; (2) 360 first-order features; (3) 440 gray-level co-occurrence matrix features; (4) 280 gray-level dependence matrix features; (5) 320 gray-level run-length matrix features; (6) 320 gray-level size zone matrix features; and (7) 100 neighboring gray-tone difference matrix features.

Method S5. Deep learning radiomics (DLR) feature extraction

Based on the 3D ROIs obtained from the CBD segmentation, the slice exhibiting the maximum cross-sectional area of the lesion was identified. Subsequently, this slice was cropped to generate a two-dimensional rectangular image encompassing the maximum extent of the lesion. Six convolutional neural networks (ResNet50,[4] DenseNet121,[5] VGG19,[6] InceptionV3,[7] MobileNet V3,[8] and ShuffleNet V2[9]) were pretrained using the ImageNet dataset, followed by transfer learning on the training set. Before training, the input images were resized to 299 × 299 pixels for InceptionV3 and 224 × 224 pixels for the remaining deep learning (DL) networks. Horizontal and vertical flipping, along with random cropping, were utilized to augment the dataset. The model parameters were updated using the stochastic gradient descent optimizer, with an initial learning rate of 0.01 that decayed according to the cosine annealing algorithm.

After comparing the output results of these six models (data not shown), we chose to use ResNet50 model for the following feature extraction. The last fully connected layer was removed, and the output value of global average pooling layer was used as DLR features. Finally, a total of 2048 DLR features from every patient were extracted through the ResNet50 model.

Method S6. WSI acquisition and preprocessing

All H&E-stained slides of CBD specimens were scanned with 40× objective lens to obtain digital WSIs. Considering the substantial scale of a WSI, each WSI was cropped into small patches measuring 512 × 512 pixels at 40× magnification level, using nonoverlapping sampling. The OnekeyAI platform was used to remove white background patches. After that, the Vahadane method, an open-source Python package for stain normalization and augmentation, was utilized to normalize the color of small patches.

Method S7. Pathological structure classification model

After preprocessing of small patches for each sample, 20 cases from center I were randomly selected, and partial patches were annotated by a pathologist with 20 years of experience. A total of 1500 patches were annotated, including 500 from the mucosal region, 500 from the

muscular layer, and 500 from the adventitial layer. These patches were randomly divided at a ratio of 3:1, with 1125 allocated for model training and the remaining 375 for internal validation. In addition, 10 cases from center II were randomly selected, and a total of 600 patches were annotated by the same pathologist for external testing. These annotations comprised 200 patches from the mucosal region, 200 from the muscular layer, and 200 from the adventitial layer.

The ResNeXt101 model,[10] which was pretrained on the ImageNet dataset, was employed for the task. During each epoch, the model's performance on the validation dataset was monitored, and the optimal epoch was determined based on the accuracy metric, utilizing early stopping to prevent overfitting. Subsequently, the pretrained model underwent fine-tuning to obtain the final structure classification model. To assess the model’s performance, macro area under the curve (AUC) was utilized as a comprehensive evaluation metric, and the “One vs. Rest” approach was applied to determine the AUC for each prediction class. Additionally, confusion matrices were employed to further scrutinize and quantify the model’s performance. Finally, the structure classification model was applied to classify the remaining unannotated patches. After that, the patches of mucosal regions were selected for subsequent analysis. This classification process provided the foundation for the second patch-level prediction strategy, which are described in Method S8.

Method S8. Two patch-level prediction strategies

Currently, weakly supervised learning is a widely utilized method in pathomics research for disease diagnosis and prediction.[11,12] Many studies have adopted a general weakly supervised approach, utilizing all patches for prediction tasks to alleviate the labor-intensive process of manually delineating ROIs on WSIs [13-15]. However, in this study, the expression of COX-2 is mainly detected in epithelial cells within the mucosal region, raising uncertainty as to whether the model’s prediction may be influenced by other unrelated regions. Consequently, we implemented and compared two separate patch-level prediction strategies. The first strategy directly utilized all preprocessed patches as inputs. In contrast, the second strategy focused solely on patches with the mucosal region, which were classified and identified using the ResNeXt101 model described above.

Six convolutional neural networks (ResNet50, DenseNet121, VGG19, InceptionV3, MobileNet V3, and ShuffleNet V2), which were pretrained on the ImageNet dataset, were applied to two patch-level prediction strategies, respectively. Each patch was labeled according to the sample it belonged to. Horizontal and vertical flipping, along with random cropping, were utilized to augment the dataset. To enhance models' adaptability to our specific task, we implemented transfer learning, using a limited dataset of the training set to adjust the weights of the models through fine-tuning. The model parameters were updated using the stochastic gradient descent optimizer with an initial learning rate set to 0.01, which decayed according to the cosine annealing algorithm.

The receiver operator characteristic (ROC) analysis and AUC were used to assess the performance of all established DL networks based on two patch-level prediction strategies, respectively. The best performing model from either of the patch-level prediction strategies was selected for further pathomics feature extraction.

Method S9. Multi-instance learning for pathomics feature extraction

Based on the best convolutional neural network of the selected approach, the patch likelihoods were aggregated in an ensemble algorithm to obtain a whole slide image (WSI)-level prediction using multi-instance learning. Two independent multi-instance learning methods were employed to aggregate the patch likelihoods, namely, the Patch Likelihood Histogram (PALHI) pipeline and Bag of Words (BoW) pipeline, which were inspired by the histogram-based method and the vocabulary-based method, respectively. In PALHI, a histogram of the occurrence of the patch likelihood was applied to represent the WSI. In BoW, each patch was mapped to a TF-IDF floating-point variable, and a TF-IDF feature vector was computed to represent the WSI.

Through the implementation of the two independent pipelines, we effectively integrated the initially dispersed patch-level predictions to produce WSI-level pathomics features for every sample.

Supplementary References

1. Kamisawa T, Ando H, Hamada Y, Fujii H, Koshinaga T, Urushihara N, et al. Diagnostic criteria for pancreaticobiliary maljunction 2013. J Hepatobiliary Pancreat Sci 2014;21:159–61.

2. Fumino S, Tokiwa K, Ono S, Iwai N. Cyclooxygenase-2 expression in the gallbladder of

patients with anomalous arrangement of the pancreaticobiliary duct. J Pediatr Surg. 2003;38:585-589.

3. van Griethuysen JJM, Fedorov A, Parmar C, et al. Computational Radiomics System to Decode the Radiographic Phenotype. Cancer Res 2017; 77: e104–7. 2.

4. He K, Zhang X, Ren S, Sun J. Deep Residual Learning for Image Recognition. Proceedings of the IEEE Conference on Computer Vision and Pattern Recognition. 2016; 770–778.

5. Huang G, Liu Z, Van Der Maaten L, Weinberger KQ. Densely Connected Convolutional Networks. Proceedings of the IEEE Conference on Computer Vision and Pattern Recognition. 2017; 4700–4708.

6. Simonyan K, Zisserman A. Very deep convolutional networks for large-scale image recognition. arXiv 1409.1556 [preprint] https://arxiv.org/abs/1409.1556. Posted September 4, 2014. Accessed September 2022.

7. Szegedy C, Vanhoucke V, Ioffe S, Shlens J, Wojna Z. Rethinking the inception architecture for computer vision. arXiv 1512.00567 [preprint] https://arxiv.org/abs/1512.00567. Posted December 2, 2015. Accessed January 9, 2023.

8. Howard AG, Zhu M, Chen B, et al. MobileNets: efficient convolutional neural networks for mobile vision applications. arXiv 1704.04861 [preprint] https://arxiv.org/abs/1704.04861. Posted April 17, 2017. Accessed September 2022.

9. Ma N, Zhang X, Zheng H-T, Sun J. ShuffleNet V2: Practical Guidelines for Efficient CNN Architecture Design. 2018; published online July 30.

10. Touvron H, Vedaldi A, Douze M, Jégou H. Fixing the train-test resolution discrepancy: FixEfficientNet. 2020.

11. Ghaffari Laleh N, Muti HS, Loeffler CML, Echle A, Saldanha OL, Mahmood F, et al. Benchmarking weakly-supervised deep learning pipelines for whole slide classification in computational pathology. Med Image Anal 2022;79:102474.

12. Cai X, Zhang H, Wang Y, Zhang J, Li T. Digital pathology-based artificial intelligence models for differential diagnosis and prognosis of sporadic odontogenic keratocysts. Int J Oral Sci 2024;16:16.

13. Schrammen PL, Ghaffari Laleh N, Echle A, et al. Weakly supervised annotation-free cancer detection and prediction of genotype in routine histopathology. J Pathol. 2022;256(1):50-60.

14. Cai X, Li L, Yu F, et al. Development of a Pathomics-Based Model for the Prediction of Malignant Transformation in Oral Leukoplakia. Lab Invest. 2023;103(8):100173.
15. Hu K, Wu Y, Huang Y, Zhou M, Wang Y, Huang X. Annotation-free deep learning algorithm trained on hematoxylin & eosin images predicts epithelial-to-mesenchymal transition phenotype and endocrine response in estrogen receptor-positive breast cancer. Breast Cancer Res. 2025;27(1):6.

Supplementary Results

Result S1. Performance of the pathological structure classification model

As shown in Supplementary Figure S2, the ResNeXt101 model demonstrated the excellent classification outcomes, with macro AUCs ranging from 0.965 to 0.984 in internal validation and external testing of the annotated patch datasets. The AUC values for identifying patches with the mucosal region were 0.986 (95% CI, 0.975–0.995) in the model's internal validation and 0.966 (95% CI, 0.948–0.980) in its external testing. This structure classification model also exhibited satisfactory performance in identifying patches with the muscular layer (AUC values ranging from 0.965 to 0.986) and the adventitial layer (AUC values ranging from 0.963 to 0.981). Further, confusion matrices visually interpreted the model's classification performance (Figure S2). Given these promising results, we concluded that the developed ResNeXt101 model could accurately identify patches with the mucosal region. Consequently, it was applied to classify the remaining unannotated patches.

Result S2. Performance of two patch-level prediction strategies

Six different convolutional neural networks, including ResNet50, DenseNet121, VGG19, InceptionV3, MobileNet V3, and ShuffleNet V2, were individually applied to the two patch-level prediction strategies and compared using the same data sets. As shown in Supplementary Figure S3, using the first weakly supervised strategy, the InceptionV3 model achieved the highest AUC of 0.622 (95% CI, 0.620–0.623) in the internal test set, whereas the DenseNet121 model led the external test set with an AUC of 0.569 (95% CI, 0.566–0.571). In contrast, when adopting the second strategy, which focused on patches with the mucosal region, the

DenseNet121 model exhibited the best performance. It achieved the highest patch-level prediction results, with an AUC of 0.710 (95% CI, 0.706–0.714) in the internal test set and 0.644 (95% CI, 0.636–0.652) in the external test set.

Supplementary Figures

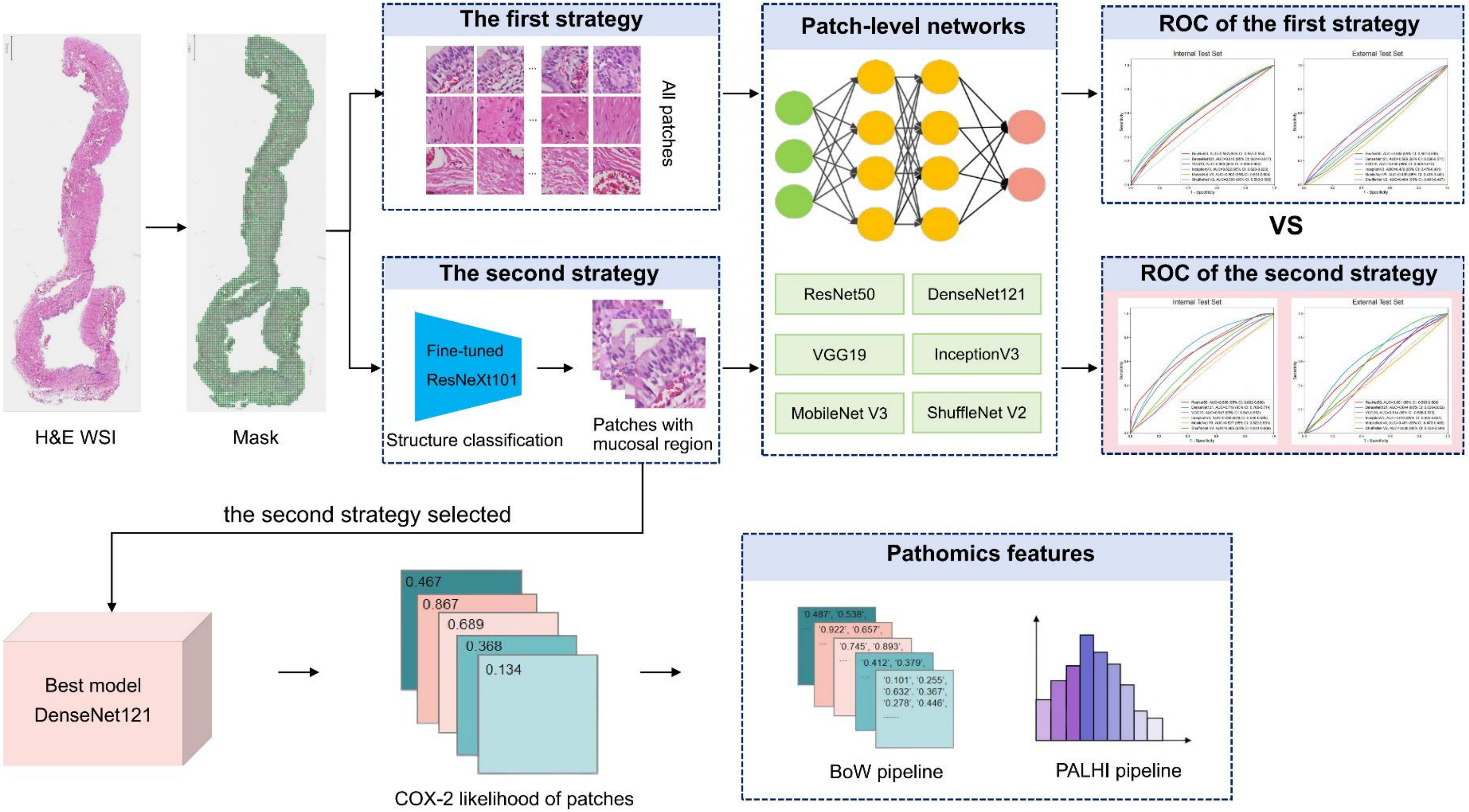

Figure S1. Pathomics workflow of the study. BoW indicates Bag of Words; COX-2, cyclooxygenase-2; H&E, hematoxylin and eosin; PALHI, Patch Likelihood Histogram; ROC, receiver operating characteristic; WSI, whole slide imaging.

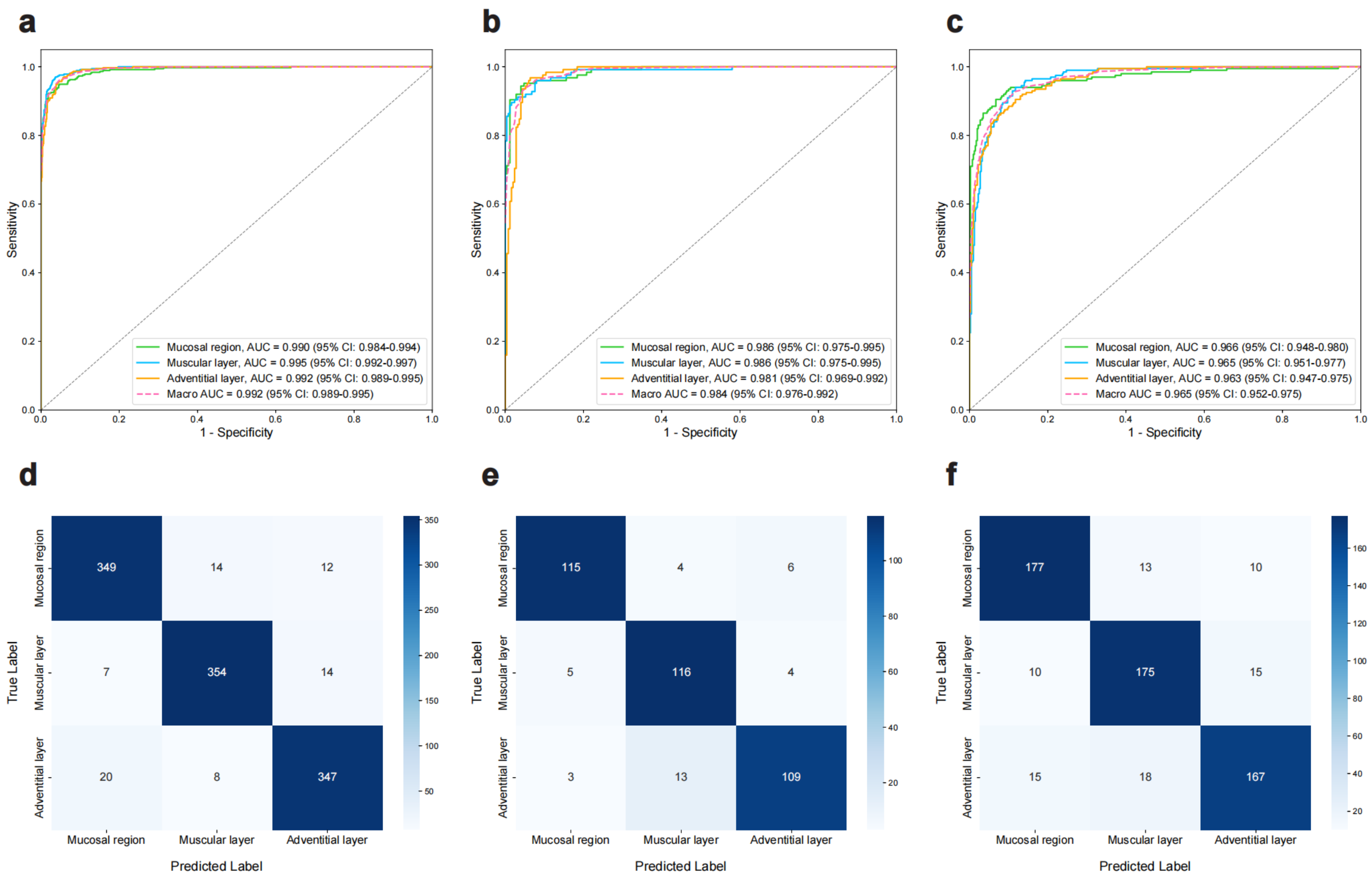

Figure S2. Receiver operating characteristic (ROC) curves for the pathological structure classification model in training (**a**), internal validation (**b**), and external testing (**c**). Confusion matrices of this model in training (**d**), internal validation (**e**), and external testing (**f**) of the annotated patch datasets.

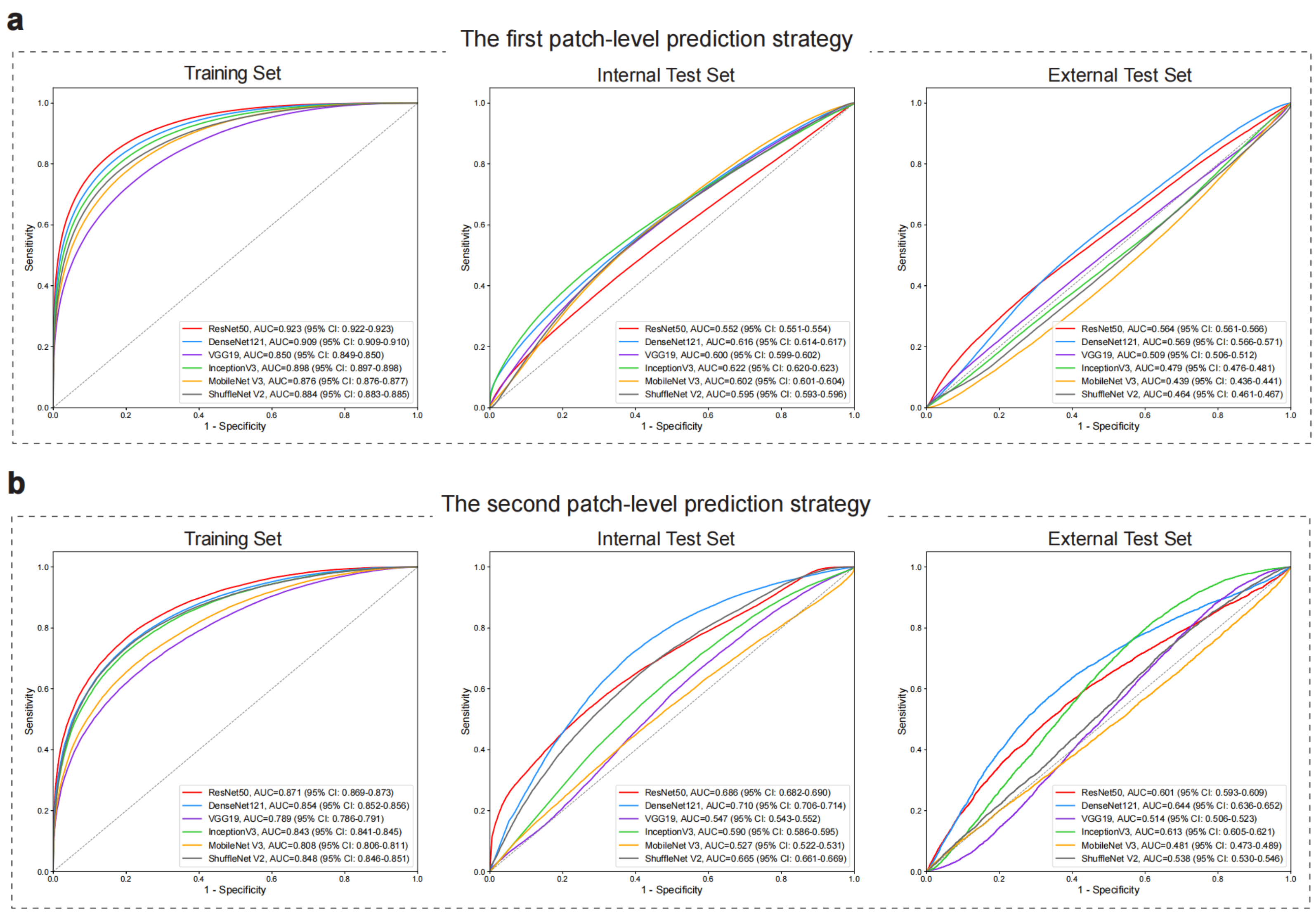

Figure S3. Receiver operating characteristic (ROC) curves of two patch-level prediction strategies respectively using six convolutional neural networks. AUC indicates area under the curve; CI, confidence interval.

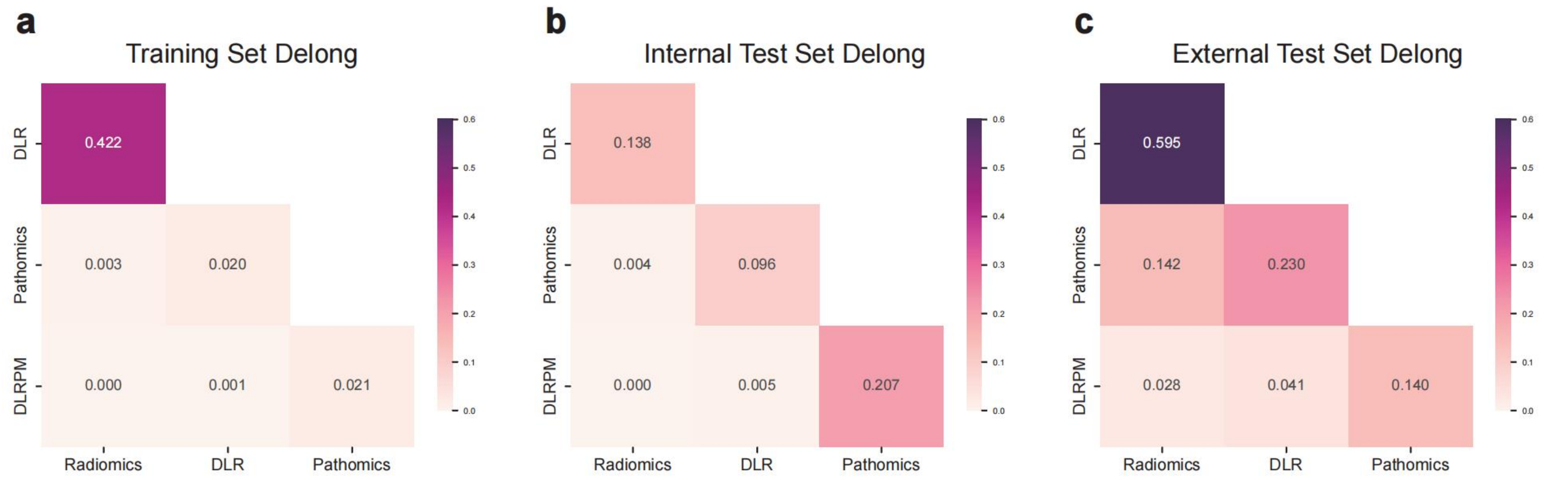

Figure S4. The heatmap of the Delong test *p* value for radiomics model, deep learning radiomics (DLR) model, pathomics model, and deep learning radiopathomics model (DLRPM) in the training set (**a**), internal test set (**b**), and external test set (**c**).

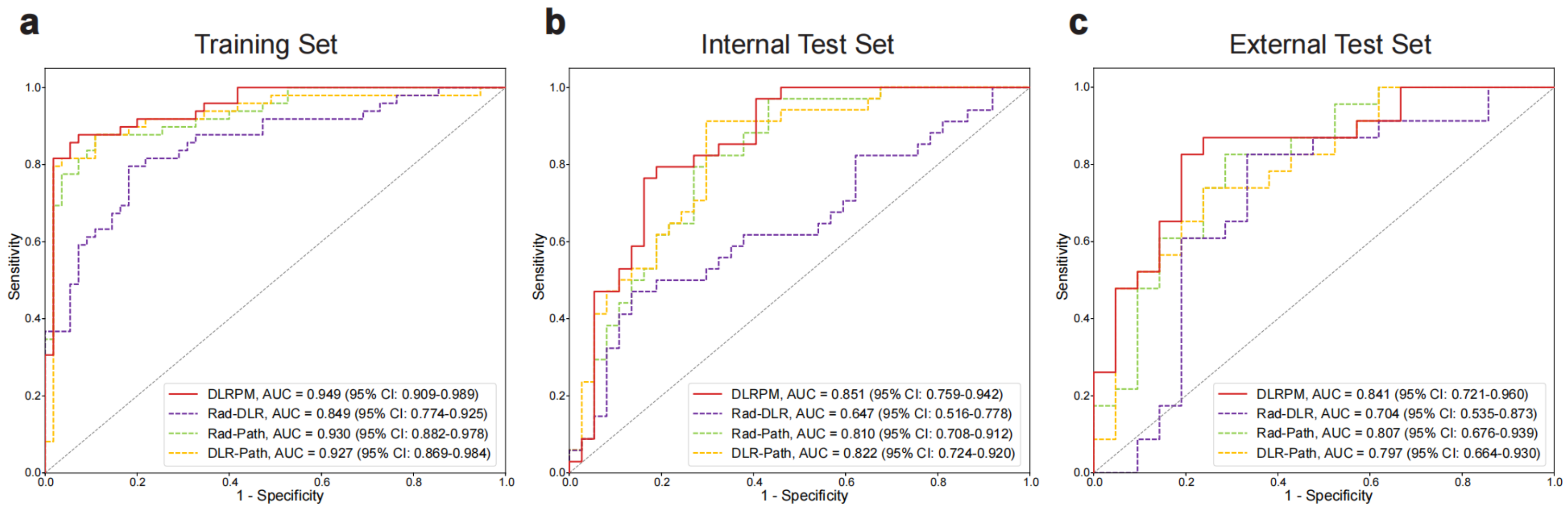

Figure S5. Receiver operating characteristic (ROC) curves of dual-modality models and deep learning radiopathomics model (DLRPM). ROC curves for predicting cyclooxygenase-2 status in pediatric patients with pancreaticobiliary maljunction among the Rad-DLR model, Rad-Path model, DLR-Path model, and DLRPM in the training set (**a**), internal test set (**b**), and external test set (**c**), respectively. AUC indicates area under the curve; CI, confidence interval.

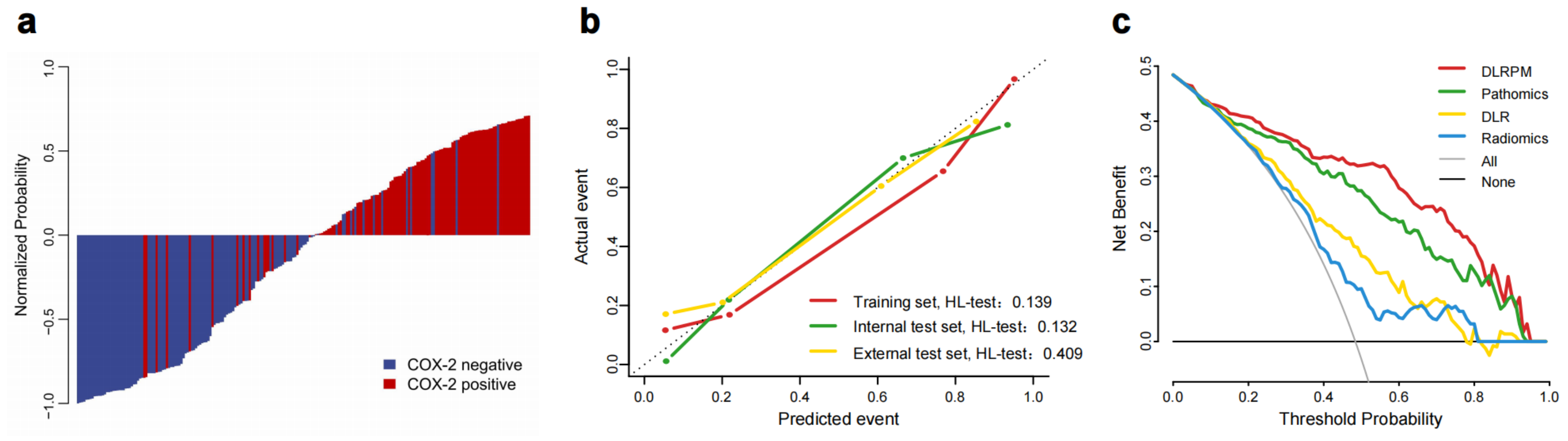

Figure S6. Deep learning radiopathomics model (DLRPM) and its performance. (**a**) Waterfall plot of each patient's relative distances from the normalized cutoff value of DLRPM in the whole datasets. (**b**) Calibration curves of DLRPM in the three data sets. (**c**) Decision curve analysis for the DLRPM, pathomics model, deep learning radiomics (DLR) model, and radiomics model. COX-2 indicates cyclooxygenase-2; HL-test, Hosmer-Lemeshow test.

Supplementary Tables

Table S1. Baseline characteristics of patients in the training, internal test, and external test sets

Characteristics	Training set (n = 104)	Internal test set (n = 71)	External test set (n = 44)	*p* value
Age (y), median (IQR)	2.8 (1.5–4.7)	2.2 (1.0–4.4)	3.4 (1.3–5.1)	0.352
Female, No. (%)	78 (75.0)	59 (83.1)	29 (65.9)	0.108
Abdominal pain, No. (%)	66 (63.5)	47 (66.2)	27 (61.4)	0.863
Jaundice, No. (%)	25 (24.0)	18 (25.4)	12 (27.3)	0.916
Fever, No. (%)	21 (20.2)	11 (15.5)	7 (15.9)	0.680
Vomiting, No. (%)	56 (53.8)	35 (49.3)	29 (65.9)	0.212
Abdominal mass, No. (%)	6 (5.8)	7 (9.9)	3 (6.8)	0.599
IVa of Todani classifcation, No. (%)	48 (46.2)	38 (53.5)	17 (38.6)	0.290
Diameter of CBD (mm), median (IQR)	22.5 (15.2–31.9)	24.9 (17.0–39.3)	22.5 (18.7–34.3)	0.396
Biliary stones, No. (%)	50 (48.1)	37 (52.1)	26 (59.1)	0.469
Peribiliary fluid collection, No. (%)	32 (30.8)	24 (33.8)	11 (25.0)	0.608
Elevated WBC count, No. (%)	31 (29.8)	25 (35.2)	10 (22.7)	0.364
Elevated AST, No. (%)	32 (30.8)	20 (28.2)	18 (40.9)	0.340
Elevated ALT, No. (%)	39 (37.5)	33 (46.5)	23 (52.3)	0.206
Elevated GGT, No. (%)	56 (53.8)	48 (67.6)	32 (72.7)	0.049
Elevated TBil, No. (%)	37 (35.6)	21 (29.6)	12 (27.3)	0.534
Elevated serum amylase, No. (%)	25 (24.0)	11 (15.5)	8 (18.2)	0.360

Abbreviation: ALT alanine aminotransferase; AST, aspartate aminotransferase; CBD, common bile duct; GGT gamma-glutamyl transferase; IQR, interquartile

range; TBil, total bilirubin; WBC, white blood cell.

Table S2. The CT imaging protocols of the two centers

Parameter	Center I	Center II
CT system	Optima CT660 (GE Healthcare, USA)	Revolution CT (GE Healthcare, USA)
Tube voltage	80–120 kVp	100–120 kVp
Tube current	Automatic tube-current	Automatic tube-current
Rotation time	0.6 s	0.5 s
Detector collimation	64×0.625 mm	256×0.625 mm
Scan thickness	5 mm	5 mm
Scan interval	5 mm	5 mm
Image matrix	512×512	512×512
Field of view	320×320 mm	350×350 mm
Contrast agent type	Omnipaque	Iodixanol
Contrast agent dosage	1.5–2.0 ml/kg	1.0–2.0 ml/kg
Contrast agent infused rate	1.0–2.0ml/s	1.0–2.5ml/s
Portal venous-phase scan	55–60 s after injection	55 s after injection
Reconstruction image thickness	0.625–1.25 mm	1.25 mm
Reconstruction image interval	0.625–1.25 mm	1.25 mm

Table S3. NRI test for prediction improvements of DLRPM compared to single-modality models in three data sets

Model	Training set		Internal test set		External test set	
	NRI (95% CI)	*p* value	NRI (95% CI)	*p* value	NRI (95% CI)	*p* value
DLRPM	/	/	/	/	/	/
Radiomics model	0.531 (0.291–0.714)	< 0.001	0.471 (0.200–0.710)	< 0.001	0.596 (0.275–0.848)	< 0.001
DLR model	0.286 (0.095–0.499)	0.003	0.393 (0.140–0.637)	< 0.001	0.369 (0.008–0.730)	0.024
Pathomics model	0.077 (0.022–0.206)	0.017	0.321 (0.110–0.522)	0.002	0.186 (0.048–0.424)	0.016

Abbreviation: CI, confidence interval; DLR, deep learning radiomics; DLRPM, deep learning radiopathomics model; NRI, net reclassification index.

Table S4. IDI test for prediction improvements of DLRPM compared to single-modality models in three data sets

Model	Training set		Internal test set		External test set	
	IDI (95% CI)	*p* value	IDI (95% CI)	*p* value	IDI (95% CI)	*p* value
DLRPM	/	/	/	/	/	/
Radiomics model	0.526 (0.421–0.619)	< 0.001	0.219 (0.133–0.288)	< 0.001	0.255 (0.113–0.385)	< 0.001
DLR model	0.474 (0.370–0.560)	< 0.001	0.162 (0.071–0.231)	< 0.001	0.229 (0.118–0.329)	< 0.001
Pathomics model	0.149 (0.118–0.182)	< 0.001	0.088 (0.045–0.131)	< 0.001	0.092 (0.034–0.141)	< 0.001

Abbreviation: CI, confidence interval; DLR, deep learning radiomics; DLRPM, deep learning radiopathomics model; IDI, integrated discrimination improvement.
